# The prevalence and causes of visual impairment in young Turkish men

**DOI:** 10.12669/pjms.314.7324

**Published:** 2015

**Authors:** Fatih Cakir Gundogan, Necmettin Kocak, Ramazan Akyildiz, Umit Yolcu, Abdullah Ilhan, Ibrahim Aydin, Selim Kilic

**Affiliations:** 1 Fatih Cakir Gundogan, Assistant Professor, Department of Ophthalmology, GATA Military Medical School, Ankara, Turkey; 2 Necmettin Kocak, MD Turkish Coast Guard Command, Health Care Department, Ankara, Turkey; 3 Ramazan Akyildiz, MD Turkish Coast Guard Command, Health Care Department, Ankara, Turkey; 4 Umit Yolcu, MD Sarikamis Military Hospital, Department of Ophthalmology, Siirt, Turkey; 5 Abdullah Ilhan, MD Erzurum Military Hospital, Department of Ophthalmology, Erzurum, Turkey; 6 Ibrahim Aydin, MD Sarikamis Military Hospital, Department of Biochemistry, Turkey; 7 Selim Kilic, Professor, Department of Public Health, GATA Military Medical School, Ankara, Turkey

**Keywords:** Visual impairment, High refractive errors, Nonsurgical retina, Optic nerve diseases

## Abstract

**Objective:**

To explore the causes and prevalence of visual impairment in young Turkish men.

**Methods:**

The health examination data of the candidates that are saved in National Defense Ministry of Turkey was used. The data of the candidates examined between 1 January 2009 and 31 December 2011 were evaluated. The total number of the candidates was 1777500. The candidates requiring advanced examination are referred to secondary and tertiary examination hospitals.

**Results:**

Fourteen thousand eight hundred sixty two(14862) out of 1777500 candidates were declared unfit for compulsory military service because of ophthalmic causes. The prevalence of ophthalmologic diseases causing unfitness for military service was found 0.746% for 2009, 0.871% for 2010 and 0.889% for 2011. These included high refractive errors which was the most frequent pathology causing unfitness (40.1%). Nonsurgical retina, vitreous and optic nerve diseases were the most frequent cause of visual impairment (0.212%). Corneal and lens pathologies were the second most frequent cause of blindness (0.101%).

**Conclusions:**

The data bank in National Defense Ministry analyzed in this study is not directly intended to explore the causes and prevalence of visual impairment in Turkey. However this study gives considerable knowledge about the causes and prevalence of visual impairment in Turkey.

## INTRODUCTION

Visual impairment has significant effects on life quality and leads to serious social and economic implications for family and society. It is also a challenging issue for employment and recruitment in many countries. Visual impairment may affect productivity and may lead to injuries. The consequences of visual impairment are an important public health issue with greater impact in the developing countries, where 80% of the world blindness occurs.^1^ The World Health Organization (WHO) encourages all countries to monitor the magnitude and causes of visual impairment in order to scrutinize and eliminate avoidable blindness.^2^ According to WHO estimates, every 5 seconds one person goes blind in the world. There were 40 to 45 million blind individuals worldwide in 2004 and about three times this figure suffered from visual impairment.^3^ It is estimated that the number of blind people worldwide will reach 76 million in 2020.^3^,^4^

Several studies reporting the causes and prevalence of blindness and visual loss exist in the literature. However, only a limited number of these reports is based on blindness registries, mainly in developed countries. Visual problems have also significant implications in the military in terms of exemption from the military service.^5^ As a developing country, Turkey is one of the biggest countries in Europe. The population of Turkey is approximately 77 million in 2013.^6^ Comparing to other European countries, Turkey has a considerably young population. According to Turkish Statistical Institute, the number of people between the age of 20-40 years-old is 25 million (32,5%).^6^ The Ministry of Health in Turkey does not have a detailed blindness registry. However, because of the compulsory military service in Turkey, each male at the age of 20-years old is examined in Military Recruiting Offices and in the military hospitals. The health status and diseases of every single young man is stored in the database of National Defense Ministry. Those data give valuable information about the prevalence of diseases and causes of disabilities. Detailed results related to musculoskeletal diseases were published previously.^7^

This study has been carried out to explore the prevalence and causes of blindness in young Turkish men who have undergone medical examination for compulsory military service between 1 January 2009 and 31 December 2011.

## METHODS

The number of young men examined for compulsory military service between 2009 and 2011 was 1.777.500. The number of people used in prevalence calculation in this study was 579.503 for 2009, 583.299 for 2010 and 614.698 for 2011.^7^

Military service is compulsory for men in Turkey. Each male at the age of 19-years old is primarily examined in Recruiting Offices where two physicians are available in accordance with Health Ability Regulation document (HARD) of Turkish Armed Forces (TAF) before they start their military service. In case of obligatory reasons, the examinations are performed after the age of 19-year old. During these examinations, disabilities that will prevent from performing military service according to HARD are analyzed. From ophthalmological point of view, proceedings for cases such as enucleation and evisceration are made in Recruiting Offices, whereas cases requiring ophthalmic examination are referred to primary military hospitals, the number of which is 37. If required, the candidates are referred to one of 6 secondary Military Hospitals. The third referral centers are GATA Military Medical School (Ankara) and Haydarpasa GATA Military Medical School (Istanbul). Health reports belonging to the men not suitable for military service are collected in Health Department Directorate of National Defense Ministry (NDM).

### Descriptions in the Survey

Decisions regarding health conditions in TAF are made in accordance with the criteria defined in HARD. All diseases have been classified as those suitable for military service, those unfit for military service (UFMS), and those under treatment of an active disease or in the recovery period of a disease. In this report, only those unfit for military service were evaluated. Those fit for military service and those under treatment or having an active disease have been excluded.

### Ophthalmic examination methods and criteria for UFMS

From ophthalmological point of view, unilateral ophthalmic pathologies causing a visual acuity (VA) under 0.2 (VA≤0,1) and bilateral ophthalmic pathologies causing a total VA (right eye plus left eye) under 1.0 (VA≤0,9) are classified as UFMS. In addition to VA measurements, some high refractive error and anisometropia data and ocular alignment disorders are categorized as UFMS irrespective of VA criteria. These criteria are (1) The sum of high axis of both eyes over 14.00 diopters, (2) anisometropia greater than 4 diopters in hypermetropics and greater than 6 diopters in myopics (by using spherical equivalents), (3) ocular movement disorders more than ‘one gaze position in one eye or equal or more than one gaze position in both eyes, (4) visual field loss greater than 2/3 in one eye or greater than 1/2 in both eyes with regard to Humphrey visual field perimeter using central 30/2 SITA Fast program and (5) any night blinding disorder including retinitis pigmentosa, stationary night blinding disorders, etc.

The diagnosis of UFMS in candidates with previous evisceration or enucleation due to any cause could be made in the recruitment offices by general practitioners (in case the candidates documented, the surgical report of the disease and evisceration cause). Most of the diagnosis causing UFMS could be made in secondary or tertiary referral military hospitals. Best corrected visual acuity was obtained with Snellen charts. Refraction data was obtained by using auto refractometers 40 minutes after instilling 3 drops of cyclopentolate (Sikloplejin, Abdi Ibrahim, Istanbul, Turkey) with 5 minutes apart. Vertex distance was set as 12.5 mm in the autorefractometers. Globe position and eye movements, pupillary reactions, biomicroscopic and fundoscopic examinations were performed in all cases.

The candidates with nyctalopia and/or photophobia were evaluated by full-field electroretinogram (ffERG) (Roland-Consult, Wiesbaden, Germany). The diagnosis of night blinding disorders (retinitis pigmentosa, fundus albipunctatus, congenital stationary night blindness) and cone dystrophies was made by using ffERG. The candidates with macular dystrophy or macular pigmentary changes were evaluated by multifocal electroretinogram (mfERG), pattern electroretinogram (PERG) and ffERG when indicated. The candidates with suspected malingering were evaluated by pattern visual evoked potentials (PVEP) and clinical malingering examination methods that were explained in detail elsewhere.^8^ The candidates with optic disc pallor were evaluated by standard ophthalmologic examination, retinal nerve fiber layer thickness analysis, PVEP and orbital and cranial magnetic resonance imaging (MRI).

### Administrative and Ethical Permissions

The data for this study was acquired from Surgeon General Office of NDM. Ethical permission of the study was obtained from Ethics Committee of Gulhane Military Medical Academy (GMMA).

### Evaluating the data and statistical analysis

In order to examine the characteristics of geographic distribution, the provinces where the recruiting offices of the candidates are located were grouped as west, south, central, northern and eastern zoning system as prepared by Turkish Statistical Institute. Since medical examinations before military service began at the ages of 19-20, the ages of the cases have been grouped as “19, 20, 21-24, and 25 years and above” when examining the demographic characteristics. The ophthalmic pathologies responsible for the UFMS were categorized into 4 groups; (1) refractive errors, (2) strabismus and nystagmus, (3) anterior segment causes, and (4) posterior segment causes. Study data was transferred to computer and analyzed with SPSS 15.0 software.

## RESULTS

Health data of 1.777.500 military service recruits whose health examinations have been carried out between the years 2009-2011 has been reviewed. It was found that 14.862 cases had ophthalmologic diseases causing unfitness for military service. The prevalence of ophthalmologic diseases causing unfitness for military service was found 0.746% for 2009, 0.871% for 2010 and 0.889% for 2011.

The most populous age group was 19 years old (31.8%). The candidates with 25 or more years old comprised 21.8% of the candidates. The most number of the examinations (25.8%) were performed in the central region of Turkey. Detailed age and region distribution of the candidates in years 2009-2011 are presented in Table-I.

**Table-I:** Demographic data of the candidates for military service.

		2009		2010		2011		Total	
		n	%	n	%	n	%	n	%
Age groups	19	1546	35.8	1540	30.3	1632	29.9	4718	31.8
	20	648	15.0	926	18.2	1037	19.0	2611	17.6
	21-24	1208	28.0	1463	28.8	1602	29.3	4273	28.8
	25 and above	919	21.2	1149	22.7	1192	21.8	3260	21.8
Region	West	739	17,1	901	17,7	920	16,8	2560	17,2
	South	426	9,9	568	11,2	552	10,1	1546	10,4
	Central	1177	27,2	1328	26,2	1329	24,3	3834	25,8
	North	631	14,6	689	13,6	776	14,2	2096	14,1
	East	1348	31,2	1592	31,4	1886	34,5	4826	32,5
Total		4321	100.0	5078	100.0	5463	100.0	14862	100.0

Among ophthalmic causes, refractive errors were the most frequent pathology causing UFMS (40.1%). Among refractive errors, ‘high axis over 14.00 diopters’ was the most common refractive error type (65.58%). Mean prevalence of refractive errors causing UFMS was 3.35 ‰. Detailed refractive error data is presented in Table-II.

**Table-II:** The distribution of ophthalmic causes of unfitness for military service.

Diagnoses	2009			2010			2011			Total		
	n	%	%*	n	%	%*	n	%	%*	n	%	%*
**Refractive Errors**	1664	38.5	0.287	2005	39.5	0.344	2292	42.0	0.373	5961	40.1	0.335
High axis over 14 dpt. (OD+OS) (any VA)	1305	30.3	0.225	1607	31.6	0.276	1817	33.3	0.296	4729	31.8	0.266
Anisometropia (VA≤0.1)	359	8.2	0.062	398	7.9	0.068	475	8.7	0.077	1232	8.3	0.069
**Ocular Alignment Disorders**	596	13.8	0.102	851	16.6	0.146	814	14.9	0.133	2261	15.3	0.128
Strabismus	233	5.4	0.040	344	6.7	0.059	338	6.1	0.055	915	6.2	0.052
a. Strabismic amblyopia (VA≤0.1)	211	4.9	0.037	300	5.9	0.051	303	5.5	0.049	814	5.5	0.046
b. EOM paralysis (any VA)	22	0.5	0.003	44	0.8	0.008	35	0.6	0.006	101	0.7	0.006
Nystagmus (VA≤0.1)	130	3.0	0.022	163	3.2	0.028	138	2.7	0.023	431	2.9	0.024
**Anterior Segment Pathologies**	859	19.8	0.148	984	19.3	0.169	1133	19.8	0.184	2975	20.1	0.167
Corneal and lens pathologies (VA≤0.1)	532	12.3	0.092	595	11.7	0.102	661	12.1	0.107	1788	12.0	0.101
Pseudophakia (VA≤0.1)	117	2.7	0.020	157	3.0	0.027	162	3.1	0.027	436	2.9	0.025
Aphakia (any VA)	187	4.3	0.032	206	4.0	0.035	199	3.6	0.032	592	4.0	0.033
Previous penetrating keratoplasty (VA≤0.1)	13	0.3	0.002	17	0.4	0.003	105	0.9	0.017	134	1.0	0.007
Previous keratorefractive operations (VA≤0.1)	10	0.2	0.002	9	0.2	0.002	6	0.1	0.001	25	0.2	0.001
**Posterior Segment Pathologies**	1384	31.9	0.294	1540	30.5	0.265	1482	27.1	0.241	4406	29.69	0.249
Previous vitrectomy surgery due to any cause and history of retinal detachment	96	2.2	0.017	116	2.3	0.020	114	2,0	0.018	326	2.19	0.019
Nonsurgical retina, vitreous and optic nerve disease	1194	27.6	0.261	1293	25.6	0.222	1264	23,3	0.206	3751	25,3	0.212
Ocular albinism	71	1.6	0.012	96	1.9	0.017	67	1.2	0.011	234	1.6	0.013
Glaucoma (due to VA ≤0.1 or VF loss)	23	0.5	0.004	35	0.7	0.006	37	0.6	0.006	95	0.6	0.005

Ocular alignment disorders (including strabismus and nystagmus) comprised 9.1% of the ophthalmic causes of UFMS. Strabismic amblyopia was the most common pathology among ocular alignment disorders (60.4%). The prevalence of strabismic amblyopia (blindness, VA≤0, 1 was 0.046%. Nystagmus comprised 2.9% of ophthalmic causes of UFMS. The mean prevalence of nystagmus causing UFMS was 0.024%. Detailed ocular alignment disorders data is seen in Table-II.

*Cornea and lens pathologies were evaluated under the title of anterior segment pathologies. Corneal and lens pathologies other than pseudophakia, aphakia and penetrating keratoplasty comprised of 12% of ophthalmic causes of UFMS. Although detailed data is not available, most of those causes of anterior segment pathologies are corneal scars and cataract. Mean prevalence of these pathologies was 0.101%. Aphakia comprised 4.0% of ophthalmic causes. The mean prevalence of aphakia was 0.033%. Pseudophakia comprised 2.9% of ophthalmic causes and the mean prevalence of pseudophakia was 0.025%. The mean prevalence of previous penetrating keratoplasty was 0.008%. Detailed anterior segment pathologies causing UFMS is presented in Table-II*.

Posterior segment pathologies comprised 31.8% of ophthalmic causes of UFMS. The mean prevalence of blindness from posterior segment pathologies was 0.266%. *Posterior segment pathologies causing UFMS is shown in Table-II*.

## DISCUSSION

To our knowledge, this is the first report using official registries in NDM on the causes of severe visual impairment from Turkey. This report includes the results of ophthalmologic examination of all young men coming up to 19-years-old in Turkey. This is because this report presents the real data-not statistics- about the causes of severe visual impairment in Turkey.

Currently, in Turkey, the overall prevalence of severe visual impairment in young men is 0.554%*. This ratio includes visual field losses (over 2/3 in an eye or over 1/2 in both eyes) related to any cause and excludes uncorrected refractive errors. Nonsurgical retina, vitreous and optic nerve diseases are the most frequent cause of visual impairment (0.212%). The detailed causes are not available in this report. The authors involved in the ophthalmic examination of the candidates for military service (FCG, UY) state that most of those causes are related to optic atrophy and retinitis pigmentosa. Corneal and lens pathologies are the second most frequent cause of blindness (0*.101%)*. Detailed causes are not available in this report. The authors also state that most of the corneal and lens pathologies causing visual impairment are related to corneal leucoma, keratoconus and cataract. The prevalence of visual impairment with pseudophakia and aphakia are 0.025% and 0.033%. The prevalence of visual impairment with previous keratorefractive operations is extremely low (0.001%). To our knowledge, there is no data available on the incidence of visual impairment in Turkey to compare with this study*.

*Refractive errors are the most prevalent cause of visual impairment in young Turkish men. The prevalence of high refractive errors is 0.335%. Visual impairment due to refractive errors is far more than that ratio as that ratio includes only the persons with refractive errors over 14.00 dpt (high axis OD+high axis OS). This report does not include the ratio of uncorrected refractive errors to all refractive errors. Weih et al. reported that 60% of participants with visual impairment due to refractive error and 82% of participants with VA of less than 20/40 to 20/60 were not wearing distance correction in a sample of Australiens.^9^ This ratio may somewhat be higher in Turkey due to lower socioeconomic status. The authors also reported that less than 1% of people aged 40-49 years had visual impairment due to uncorrected refractive error*.

*The prevalence of registered blindness was 0.17%-0.22% in Ireland (blindness was defined as BCVA in the better eye of less than 6/60)^10^ and 0.26%-0.32% in Israel (using WHO criteria).^11^ The incidence of registered blindness was 0.036%-0.029% in Israel (using WHO criteria), 0.02% in Ireland using (US criteria) and 0.012% in Southern Germany (BCVA in the better eye less than 1/50).^11^-^14^ The prevalence of blindness in a district of China in male population with an age between 1 to 29-years-old was reported as 0.015% in 2009.^14^ The reported blindness increased from 114.7 per 100.000 in 2003 to 145.8 per 100.000 in 2006 to 165.9 per 100.000 in 2009 although the population of the district remained stable. The authors stated that increase in prevalence during 2001-2009 represented increased registration rather than increasing levels of disease. The registries in NDM did not allow us to have the prevalence of blindness in terms of WHO or US criteria. However, from our data, we can state that the prevalence of monocular blindness in Turkey (VA≤0.1) is 0.055%*. This prevalence cannot be directly compared to the prevalence of blindness reported from other countries.
